# “You have to believe the patient”: What do people with fibromyalgia find helpful (and hindering) when accessing health care?

**DOI:** 10.1080/24740527.2023.2176745

**Published:** 2023-03-30

**Authors:** Ria K. Nishikawara, Izabela Z. Schultz, Lee D. Butterfield, John W. Murray

**Affiliations:** a Educational and Counselling Psychology, and Special Education, The University of British Columbia, Vancouver, British Columbia, Canada; b Vancouver, British Columbia, Canada; c North Vancouver, British Columbia, Canada; d Langley, British Columbia, Canada

**Keywords:** fibromyalgia, biopsychosocial, chronic pain, health care, enhanced critical incident technique (ECIT)

## Abstract

**Background:**

Fibromyalgia (FM) is a complex, still poorly understood, and difficult-to-treat chronic pain condition for which many people struggle to find adequate care.

**Aims:**

This study investigated the research question, “What do people accessing health care services for fibromyalgia perceive as helpful, hindering, and absent but desired?” with the aim of identifying clear, implementable changes for clinical practice.

**Materials and methods:**

This study used the enhanced critical incident technique (ECIT), a qualitative research method that focuses on helping, hindering, and desired factors, to explore the health care experiences of 14 individuals (12 women and 2 men) diagnosed with FM.

**Results:**

Using qualitative data analysis, results identified three categories of health care experiences: (1) systemic navigation, including financial and economic security; accessibility, flexibility, and continuity of care; and diversity of treatment options; (2) clinician–patient alliance, including invalidation and prejudice; therapeutic bond; and clinician–patient alignment on treatment plan; and (3) patient self-management strategies, including information-seeking and education, self-advocacy, social supports, symptom management strategies, and other coping strategies. Participants tended to conceptualize their health care concerns as a multilayered systemic problem.

**Conclusions:**

Participants described a medical system they perceived as poorly equipped to support their needs and tended to invalidate their health concerns. Helping experiences tended to be the result of unique efforts on the part of individual clinicians. Findings emphasize the importance of recognizing the complexities and psychological impact of pain, trusting clinician–patient relationships, multidisciplinary/interdisciplinary care within a biopsychosocial framework, and improved education and awareness around psychosocial aspects of FM and effective management of chronic pain.

## RÉSUMÉ

**Contexte:** La fibromyalgie est une maladie chronique complexe, encore mal comprise et difficile à traiter, pour laquelle beaucoup de gens ont du mal à trouver des soins adéquats.

**Objectifs:** Cette étude s’est penchée sur la question de recherche : « Qu’est-ce que les personnes qui ont accès aux soins de santé pour la fibromyalgie perçoivent comme utile, gênant, et absent mais désiré? » en vue de définir des changements clairs et réalisables pour la pratique clinique.

**Matériaux et méthodes:** Cette étude a utilisé la technique des incidents critiques améliorée, une méthode de recherche qualitative qui met l’accent sur les facteurs aidants, entravants et souhaités, afin d’étudier les expériences de soins de santé de 14 personnes (12 femmes et 2 hommes) ayant reçu un diagnostic de fibromyalgie.

**Résultats:** À l’aide d’une analyse qualitative des données, les résultats ont permis de définir trois catégories d’expériences de soins de santé : (1) la navigation systémique, y compris la sécurité financière et économique; l’accessibilité, la flexibilité et la continuité des soins; et la diversité des options de traitement; (2) l’alliance clinicien-patient, y compris l’invalidation et les préjugés; le lien thérapeutique; et l’alignement clinicien-patient sur le plan de traitement; et (3) les stratégies d’auto-prise en charge des patients, y compris la recherche d’information et l’éducation, l’autonomie sociale, les soutiens sociaux, les stratégies de prise en charge des symptômes et d’autres stratégies d’adaptation. Les participants avaient tendance à conceptualiser leurs préoccupations en matière de soins de santé comme un problème systémique à plusieurs niveaux.

**Conclusions:** Les participants ont décrit un système médical qu’ils percevaient comme mal outillé pour répondre à leurs besoins et qui avait tendance à invalider leurs préoccupations en matière de santé. Les expériences d’aide tendaient à être le résultat d’efforts isolés de la part de certains cliniciens. Les résultats soulignent l’importance de reconnaître la complexité et les répercussions psychologiques de la douleur, des relations de confiance entre cliniciens et patients, des soins multidisciplinaires ou interdisciplinaires dans un cadre biopsychosocial. Ils soulignent aussi l’importance d’améliorer l’éducation et la sensibilisation aux aspects psychosociaux de la fibromyalgie et de la prise en charge efficace de la douleur chronique.

## Introduction

Fibromyalgia (FM) is a complex and still poorly understood biopsychosocial condition, characterized by diffuse pain, chronic fatigue, nonrestorative sleep, and cognitive difficulties.^1^,^2^ FM is associated with co-occurring conditions including bowel and bladder difficulties, anxiety, and depression.^3^ Many people with FM state that their relationships and work suffer due to debilitating symptoms, with many reducing work hours by 50% to 75% and unemployment rates as high as 51% to 80.6%.^4^ They also face social stigmas of nonvisible disability, often followed by negative alterations in sense of self and identity.^5^

Despite significant functional and clinical impacts, people with FM frequently report feeling invalidated about the legitimacy of their symptoms because FM has no objective diagnostic markers.^6–8^ Health professionals often describe patients with FM as a challenging and discouraging population to work with.^9–11^ A mismatch in patient and clinician expectations can contribute to strain or disconnection in the clinician–patient relationship and negative health outcomes.^7,9^ These difficulties can take a psychological toll on both sides, contributing to possible emotional distress and worsening symptoms for people with FM^6,12^ and increasing the risk of clinicians feeling overwhelmed and burned out.^9,11^

### Health care for FM

People with FM experience the “double burden” of living with a debilitating condition while having its reality questioned by others.^13–15^ Lack of consensus on objectively testable symptoms has resulted in “fibromyalgia wars” over the legitimacy and clinical usefulness of the FM diagnosis, contributing to the stigmatization of FM.^10^ Thus, people with FM struggle with trying to understand their complex health condition, stigmatizing social and medical attitudes, and inconsistent guidance.^15^

Though evidence-based health care guidelines for FM have been created,^16^ substantive systemic and organizational changes are slow to come, causing care providers to face challenges with incorporating recommendations. There is growing acknowledgment of FM as best understood through a biopsychosocial framework^17^; however, sometimes this model is misinterpreted to mean that chronic pain conditions are psychogenic,^18^ which risks further stigmatization and self-stigmatization. In fact, a biopsychosocial model of chronic pain disability suggests that effective treatment considers the patient as a whole and requires comprehensive, multidisciplinary/multimodal treatment; for example, educational, psychological, and exercise therapies, alongside symptom-based pharmacologic treatments as needed.^16^

### Aims

A growing body of literature has touched on the health care experiences of people with FM.^7,15,19–23^ This study addressed the topic in a novel way, focusing on barriers and facilitators, inquiring “What do people accessing health care services for Fibromyalgia perceive as helpful, hindering, and absent but desired?” We aim to contribute tangible helping and hindering factors to the literature, uniquely from both relational and systemic perspectives, to improve support to this patient population and provide guidance and recommendations for health care providers.

Guidelines emphasize the importance of clinicians being knowledgeable about FM, as well as empathetic, collaborative, and accepting.^17^ With improved alignment between patient needs and expectations and evidence-informed clinical practices in FM, enhanced health care and functional outcomes are expected to follow. This qualitative study aims to explore health care experiences and their psychological impacts in people with FM to expand the evidentiary basis for bridging this gap.

## Materials and Methods

This study used the enhanced critical incident technique (ECIT), an exploratory qualitative research method that balances postpositivist and constructivist approaches.^24^ The ECIT expands on the critical incident technique described by Flanagan,^25^ a robust and flexible method that has been found useful in health care research for studying subjective experiences and solving practical problems.^26,27^ This method is unique in its focus on identifying *critical incidents*—factors that hinder or help the activity being studied that can be used to build descriptive categories that can inform recommendations for practice and organizational change. *Wish list items* are also identified—factors that were not present at the time of the participant’s experience but that they believe would have been helpful.^28^

### Participants

Recruitment for this study was conducted through posters, social media, and word of mouth. To be included, participants needed to be over the age of 18, with a formal diagnosis of FM. Those with co-occurring conditions associated with FM (e.g., anxiety, depression, chronic fatigue) were included. Individuals with nonassociated painful conditions (e.g., cancer) were excluded. In total, 14 individuals participated in the study. All participants were located in the Lower Mainland, British Columbia, at the time of the study; however, they described health care experiences that took place throughout Canada (primarily urban settings; Table 1).

**Table 1.** Demographic details.

Demographic characteristics	Participant information
Sex	Female = 12
	Male = 2
Age	Range = 22–76
	Mean = 48.5
	Median = 50
Years with symptoms	Range = 1.5–50+
Years with diagnosis	Range = <1–36
Work status impacted by symptoms	All
Employment status	Not working = 8
	Working/in school = 6
Education	Some college/university = 3
	Undergraduate degree = 7
	Graduate degree = 3
	Doctoral degree = 1 (currently enrolled)
Insurance	No private/employer insurance = 2
	Held private/employer insurance = 8
	Did not disclose = 2

### Data Collection

Data were collected following the procedures set out by Butterfield et al.,^28^ beginning with verbal and written informed consent. Interviews were audio-recorded, lasted between 1 and 2.5 hours, and followed a three-part protocol. Part 1 explored contextual information, wherein participants were asked about their health care journeys with FM. The definition of health care was left open, allowing participants to discuss experiences they deemed relevant. This phase also helped establish rapport through hearing participants’ stories and created context for the critical incident interview. The critical incident interview explored *helping* and *hindering incidents* and *wish list items*, with probes to elicit specific examples and the importance of each incident. Lastly, participants completed a demographics questionnaire.

Follow-ups (member checking) were conducted to confirm accuracy of the data analysis. Participants were provided a summary of critical incidents from their interviews and were asked to confirm whether these were complete and correctly understood. Participants were also asked whether they agreed with the categorization of incidents and whether they desired any changes. Ten participants responded to these cross-checks, and no changes were made at this stage.

Interviews were conducted by the first author until exhaustiveness (saturation) was reached in the data. Although saturation was reached after seven interviews, an additional seven interviews were conducted to enhance data credibility due to the heterogeneity of people with FM.

### Data Analysis

Data analysis followed the ECIT guidelines (outlined in detail in Butterfield et al.^28^). Once interviews were transcribed and anonymized, the researchers reviewed the transcripts to familiarize themselves with their content. Transcripts were then coded, and extracted incidents were grouped using an iterative process until descriptive categories were determined.

All nine credibility and trustworthiness checks from the ECIT method^28^ were used: (1) audio recording the interviews, (2) interview fidelity, (3) independent extraction of critical incidents, (4) calculating exhaustiveness, (5) calculating participation rates, (6) placement of incidents into categories by an independent judge, (7) cross-checking of categories by participants, (8) expert opinions, and (9) theoretical agreement. The study data met or exceeded the established credibility check standards.

## Results

The interviews generated a total of 618 critical incidents participants identified as hindering or helpful in their experiences of accessing health care services for FM, including 297 hindering incidents, 231 helping incidents, and 90 wish list items, which were organized into three categories, displayed in Table 2. The first column displays the title of the category, followed by the number of hindering incidents and number of participants who endorsed it (participation rate), followed by the number of helping incidents and participation rate for that category and, lastly, the number of wish list items and participation rate. Hindering incidents are displayed first because they were more frequently endorsed and emphasized in participant accounts. (Note: In ECIT, *N* indicates the number of incidents rather than participants.)

**Table 2.** Categorization of results.

Category	Hindering Incidents (HI) *N* = 297	Number of participants (% of total)	Helping Incidents (HE) *N* = 231	Number of participants (% of total)	Wish List (WL) *N* = 90	Number of participants (% of total)
Systemic navigation	100	14 (100)	66	11 (79)	44	8 (57)
(a) Financial and economic security (b) Accessibility, flexibility, and continuity of care (c) Diversity of treatment options						
Clinician–patient alliance	197	14 (100)	109	14 (100)	41	12 (86)
(a) Invalidation and prejudice (b) Therapeutic bond (c) Clinician–patient alignment on treatment plan						
Patient self-management strategies	N/A		56	14 (100)	5	4 (29)
(a) Information-seeking and education (b) Self-advocacy (c) Social supports (d) Symptom management strategies (e) Other coping strategies						

Each category is discussed below, with subthemes and participant examples for the most endorsed aspect (i.e., hindering or helping) of the category. The incidents relate to encounters with a range of clinicians across primary and specialized care and complementary and alternative medicine (CAM); however, the physician–patient relationship was most emphasized and described.

### Systemic Navigation Factors

Systemic navigation factors addressed barriers and facilitators participants encountered in accessing health care services, such as the following:

(a) *Financial and economic security*: Hindering incidents addressed financial instability or barriers, like difficulties with insurance providers or affordability of treatments.

P13: I was using my whole cheque to pay for food and rent and medical stuff.

(b) *Accessibility, flexibility, and continuity of care*: Hindering incidents addressed accessibility challenges such as inflexible cancelation policies, lack of virtual care options, scarcity of family doctors, long wait times for specialists, and lack of follow-up.

P13: It took me two years of active searching and being desperately in need of immediate care to find a GP … bounced around between walk-in clinics and the ER.

(c) *Diversity of treatment options*: Hindering incidents reflected limitations of the biomedical model of care, lack of services for chronic health concerns, and efforts to access diverse treatment methods, including comprehensive, interdisciplinary programs, as well as CAM.

P8: The lack of health care services [has been unhelpful]. The only heartening piece is that I know there’s something taking place at Women’s Hospital … but I think there’s still a long way to go.

Some also described negative experiences with CAM providers and lack of regulation of these industries.

P8: He claimed that he was an FM specialist. I went on my own dime, which should have been my first clue that something was amiss … almost as if he was taking advantage of the situation with supplements and hokey pokey treatments. Ironically, overnight he took down the shingle and disappeared.

### Clinician–patient Alliance Factors

Clinician–patient alliance factors addressed barriers and facilitators participants encountered in alliance formation with their health care providers, such as the following:

(a) *Invalidation and prejudice*: Hindering incidents addressed experiences of prejudice and invalidation from health care providers and their links with internalized stigma. Examples included prejudicial beliefs like sexism and ageism; invalidation, such as lack of understanding of FM or doubts about the existence FM, as well as minimizing or dismissing symptoms as psychogenic; and internalization, or the ways in which participants assimilated these stigmatizing messages into their self-perceptions.

P1: [My doctor] was a bit dismissive with my symptoms and the pain that I was having. … It makes you feel like you’re weak. … I’m like, “Wow, I’m not a strong person. I shouldn’t be feeling this way.”

P9: I’ve had doctors tell me they don’t think fibromyalgia is real. … I had one doctor tell me that fibromyalgia is a symptom of depression, so when you’re depressed sometimes you have increased levels of pain.

(b) *Therapeutic bond*: Helping incidents in this category described clinicians who were empathetic, collaborative, and made participants feel like they mattered.

P13: “We had a good working relationship and that made a difference. … When there’s mutual trust, when you matter to them, you do better.

Incidents occurred in a variety of health care relationships, including opportunities to deeply explore the impacts of chronic illness and learn coping skills in psychotherapy.

(c) *Clinician–patient alignment on treatment plan*: Hindering incidents in this category described misalignment between clinician and patient regarding treatment plans, including lack of informed consent, and adverse treatment effects, many of which contributed to emotional distress and erosion of trust.

P3: [My doctor] gave me a drug and didn’t explain to me what could happen with it and went away for a week. By the time she came back, I was seriously ill. I could barely stand.

When participants understood the treatment plan and agreed with the rationale, they tended to be more accepting of adverse effects. Many participants also highlighted incidents where medication and physical activity recommendations did not align with their understandings of their needs or abilities.

### Patient self-management Strategies

Helping incidents in this category reflected participants’ agentic strategies to establish structure and knowledge that supported their well-being and helped manage their symptoms, often in response to perceived gaps in care, such as (a) *information seeking and education*: personal efforts in finding research and information; (b) *self-advocacy*: steps participants took in representing their own needs and interests to their health care providers, (c) *social supports*: participants’ social networks, including friends and family, who provided emotional and practical supports; (d) *symptom management strategies*: such as mindfulness, cognitive–behavioral strategies, and pain education, that participants used to manage their syptoms; and (e) *other coping strategies*: preparation, planning, spirituality, and creative practices.

## Discussion

**Figure 1.**
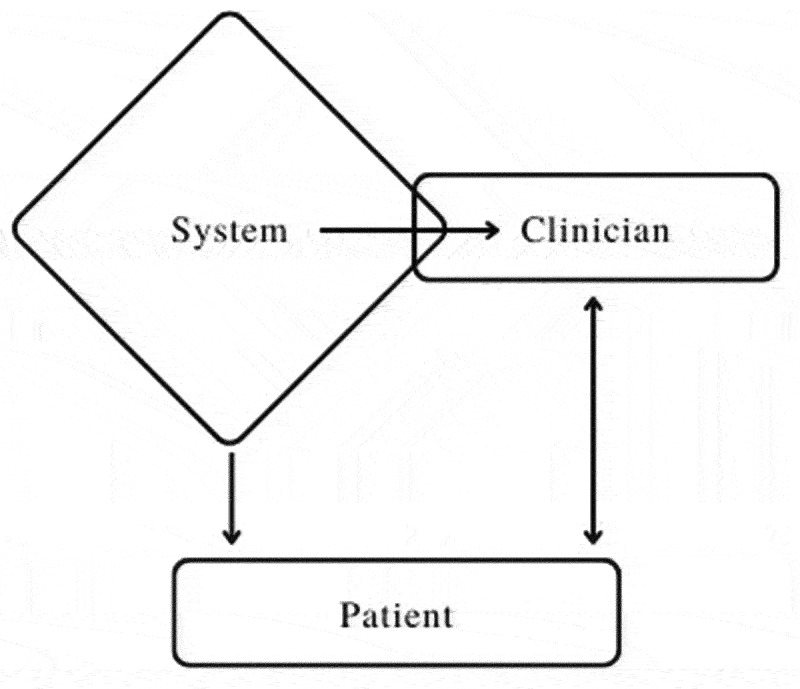
The interconnections between systemic, clinician, and patient factors. Clinicians are constrained by systemic factors but can also be seen as representing the system, which influences patients both directly and indirectly. Patients do not directly influence the system; however, clinician and patient relationships may be mutually influential.

There is a paucity of studies taking an integrative person–system approach to understanding the psychosocial complexities of the health care experience of people with FM. In this study exploring health care experiences for FM, participants addressed hindering, helping, and desired factors related to their health care relationships and perceptions of the health care system overall. The three categories of findings from this study—systemic navigation, clinician–patient alliance, and patient self-management strategies—highlight the ways in which systemic barriers influence interpersonal dynamics in health care, as well as patient sense of self and personal coping strategies to respond to perceived gaps in care. We observed significant interconnection between systemic barriers and clinician–patient alliance factors, given that clinicians are bound by systemic constraints, but may also represent that system and its constraints in a patient's eyes.. Although participants described many ways in which they were able to self-advocate and take agency within specific health care relationships, all participants in this study perceived the overall health care system as slow and averse to respond to their needs (Figure 1).

Many of our subthemes affirm findings of prior studies, further strengthening the knowledge base addressing health care experiences of people with FM through the use of a novel mode of inquiry (ECIT). Our findings contribute to the literature through presenting these diverse themes within a single study in an integrative, system–individual framework. Presenting the health care experiences of people with FM in this way may contribute to refining applications of the biopsychosocial model for this population.

Although all participants described hindering incidents in their health care interactions, most also had positive encounters with specific clinicians whom they described as helpful. We offer findings about the systemic context, insight into patients’ overall journeys with FM, and interpersonally oriented suggestions to help guide the clinician–patient relationship. A strong clinician–patient working alliance is a particularly critical component of treating nonspecific chronic pain, leading to improved patient engagement and better health outcomes.^11^

### Implications of the Study at the Systemic Level

Although many of our themes are not new to FM or chronic pain literature, this study is unique in demonstrating the extent to which participants conceptualize their negative health care experiences as a multilayered systemic problem. Not only is interdisciplinarity the recommended approach for chronic pain, but this study suggests that it is something patients are explicitly or implicitly seeking. Participants in this study echoed a need for information about FM,^29^ highlighting how few participants had received biopsychosocially framed approaches like pain education, energy conservation tools, and psychosocial interventions. A further outcome of this study emphasizes how participants employed strategies like personal research, self-advocacy, and self-management to compensate for these gaps in care. Participants in this study demonstrated strong motivation to continue to learn about their condition and strategies to improve their well-being, showing receptivity and desire for accessible and accurate information about FM.

Participants discussed encountering prejudicial beliefs and emphasized their desire for acceptance of FM as a legitimate illness deserving of care. We found support for findings related to younger participants experiencing dismissal on the assumption that they could not be dealing with such severe symptoms at their young age.^22^ One participant also described racism leading to delays in her diagnosis due to lack of understanding of how symptoms present across diverse ethnic and racial populations. Notably, this article contributes to the literature on the damaging and potentially disabling impacts of stigmatization and invalidation among people living with chronic pain; participants highlighted how stigmatizations of FM at a systemic health care level can lead to internalization of these beliefs, resulting in self-judgment and risk of self-stigmatization in the individual, potentially leading to negative mental health consequences.^30,31^

### Implications of the Study at the clinician–patient Level

Though participants acknowledged systemic barriers, they also had much to say about receptivity from individual practitioners. Many of our findings are consistent with the literature on patient-centered care: patients desire to feel connected, cared for, and listened to by health care providers.^19,32^ Participants in this study further echoed findings on the impacts of financial barriers and the importance of accounting for accessibility in FM treatment.^33^ Though individual care providers might have limited options to change systemic constraints, they can take a collaborative, patient-centered approach, clearly outlining appointment parameters, informing patients of their clinical role, clarifying patient goals, and keeping affordable options and appropriate medical and nonmedical referrals in mind.^34^ Providing accessibility options such as telehealth appointments and modularizing educational programs were also suggested.

Importantly, our study supports the following key messages for improving health care experiences in FM and helping bridge the gap between patient expectations and reality of service delivery:

1. Research shows that clinicians are more likely to discount symptoms, are less likely to provide medical intervention, and are more likely to attribute symptoms to psychosocial factors when patients report high levels of pain.^35–37^ It may be helpful for health care providers to understand the complexities and psychological impact of patients’ health care experiences and take a stance of accepting their patients’ experiences as real rather than questioning the extent and “objective evidence” of their suffering.
2. A trusting relationship between patient and provider impacts health outcomes from both psychological and functional outcome perspectives. Participants emphasized the importance of strong relationships with their providers, as well as informed consent, collaborative treatment planning, and continuity of care.
3. Individuals with persistent pain need validation of their experiences of pain, while also learning that there are many factors that contribute to pain that can be managed with multidisciplinary/interdisciplinary care. Where clinicians are not in a position to provide pain education, they might offer to share referrals or resources (e.g., Pain Care U, Tame the Beast).
4. Improved education and awareness around psychosocial aspects of FM and health impacts of patient health care experiences may be needed to guide providers around effective management of chronic pain, building trusting clinician–patient relationships, and selecting the most appropriate combination of pharmacologic and nonpharmacologic treatment approaches.^38^ Clinicians are also encouraged to learn about nociplastic pain.^39^ Though many clinicians may not have received specific medical training in these areas, research and professional development initiatives have emerged around chronic and nociplastic pain conditions to support clinicians working with these patients (e.g., Pain BC, International Association for the Study of Pain, Neuro Orthopaedic Institute (NOI group)).

### Limitations and Recommendations for Further Research

As is common in interview studies, we recognize that self-selection of participants can contribute bias to findings, such as the possibility that individuals with notable challenges in their health care experiences may be more drawn to participate. Further, all participants had postsecondary education, and most identified as women. Demographic data regarding race and ethnicity were not collected in this study, which is critical to collect in future studies. Continued research with diverse populations is recommended to understand the needs of a broader range of individuals with FM.

The definition of health care was left open to participants. Varied definitions may be a limitation in this study; however, they also depict how complex health care systems can be to consumers. Most responses focused on primary care and public pain programs, though many incidents addressed CAM treatments.

The experiences described in this study took place over a broad span of time and before COVID-19; therefore, these descriptions may not all pertain to the current state of health care service provision for FM. It is anticipated that challenges experienced by persons with FM will be exacerbated when health care capacity hovers around its limits, as it does now due to the pandemic.

### Conclusions

Recognition of the complex relationships among pain, psychosocial factors, systemic contexts, and patient functioning and their ability to interact in a cyclical and reciprocal manner in a health care context can help guide treatment for FM. The importance of modifiable factors like strong therapeutic alliance and informed consent needs to be addressed to improve future health care outcomes in FM and prevent needless distress, adverse treatment outcomes, self-stigmatization, and potential negative mental health impacts. Further, clinicians should be encouraged to advocate where possible toward broader systemic change that will benefit all but especially those with poorly understood biopsychosocial conditions, such as FM, who are at risk for chronic disability.

This action can include turning a biopsychosocial treatment model into the interdisciplinary service reality through advocating for training and professional development in (1) understanding the complexity and modifiability of impact on patient experiences within health care, (2) awareness of psychosocial aspects of chronic pain and disability, (3) enhancement of interpersonal skills that improve effectiveness of clinician–patient relationships, and (4) how to achieve interdisciplinarity in treatment of chronic pain in the current health care context.

Living with chronic pain impacts virtually every aspect of life, including energy, sleep, and mood, not to mention health care access, financial losses, as well as friend, family, work, and romantic relationships.^40^ All of these issues and more can lead to complex and painful feelings that health care professionals may not believe they have the time or the training to adequately respond to. That said, participants highlighted that even brief encounters can be meaningful and validating, such as clinicians articulating that they believe the patient, that they acknowledge their pain is real, and that they want to help. Though systemic change is an ongoing process, our findings suggest that small and incremental changes are likely to make an important difference and can produce more rewarding interactions on both sides, improving health care experiences and outcomes of people with FM, while contributing to disability prevention.

## References

[1] Okifuji A, Bradshaw DH, Donaldson GW, Turk DC. Sequential analyses of daily symptoms in women with fibromyalgia syndrome. J Pain. 2011;12:84–9. 20591745 10.1016/j.jpain.2010.05.003 PMC2980792

[2] Wolfe F, Häuser W. Fibromyalgia diagnosis and diagnostic criteria. Ann Med. 2011;43:495–502. 21770697 10.3109/07853890.2011.595734

[3] International Association for the Study of Pain. Fibromyalgia syndrome: prevalent and perplexing. Pain Clin 2003;11:3.

[4] Skaer TL. Fibromyalgia: disease synopsis, medication cost effectiveness and economic burden. PharmacoEconomics 2014;32:457–66. doi:10.1007/s40273-014-0137-y. 24504852

[5] Schultz I, Stowel AW, Feuerstein M, Gatchel R. Models of return to work for musculoskeletal disorders. J Occup Rehabil. 2007;17:327–53. 17286211 10.1007/s10926-007-9071-6

[6] McMahon L, Murray C, Sanderson J, Daiches A. “Governed by the pain”: narratives of fibromyalgia. Disabil Rehabil. 2012;34(16):1358–66. doi:10.3109/09638288.2011.645114. 22263672

[7] Briones-Vozmediano E, Vives-Cases C, Ronda-Pérez E, Gil-González D. Patients’ and professionals’ views on managing fibromyalgia. Pain Research & Management. 2013;18(1):19–24. doi:10.1155/2013/742510. 23457682 PMC3665433

[8] Madden S, Sim J. Creating meaning in fibromyalgia syndrome. Soc Sci Med. 2006;63(11):2962–73. doi:10.1016/j.socscimed.2006.06.020. 16949713

[9] Asbring P, Narvanen A-L. Ideal versus reality: physicians [sic] perspectives on patients with chronic fatigue syndrome (CFS) and fibromyalgia. Soc Sci Med. 2003;57(4):711–20. doi:10.1016/S0277-9536(02). 12821018

[10] Briones-Vozmediano E, Ohman A, Goicolea I, Vives-Cases C. “The complaining women”: health professionals’ perceptions on patients with fibromyalgia in Spain. Disabil Rehabil. 2018;4(14):1679–85. doi:10.1080/09638288.2017.1306759. 28385050

[11] Matthias MS, Parpart AL, Nyland KA, Huffman MA, Stubbs DL, Sargen C, Bair MJ. The patient-provider relationship in chronic pain care: providers’ perspectives. Pain Med 2010;11:1688–97. 21044259 10.1111/j.1526-4637.2010.00980.x

[12] Juuso P, Skär L, Olsson M, Söderberg S. Meanings of being received and met by others as experienced by women with fibromyalgia. Qual Health Res. 2014;24(10):1381–90. doi:10.1177/1049732314547540. 25147217

[13] Juuso P, Skar L, Olsson M, Soderberg S. Living with a double burden: meanings of pain for women with fibromyalgia. Int J Qual Stud Health Well-being. 2011;6:7184–93. 10.3402/qhw.v6i3.7184 PMC3136954 21765861

[14] Yaghmaian R, Smedema SM. A feminist, biopsychosocial subjective well-being framework for women with Fibromyalgia. Rehabil Psychol. 2019;64(2):154–66. doi:10.1037/rep0000226. 30265038

[15] Ashe SC, Furness PJ, Taylor SJ, Yahwood-Small S, Lawson K. A qualitative exploration of the experiences of living with and being treated for fibromyalgia. Health Psychol Open 2017;4:2. 10.1177/2055102917724336 PMC5779932 29379616

[16] Fitzcharles M, Ste-Marie PA, Goldenberg DL, Pereira JX, Abbey S, Choinière M, Ko G, Moulin D, Panopalis P, Shir Y 2012. The Canadian guidelines for the diagnosis and management of fibromyalgia syndrome; [accessed July 23, 2022]. http://fmguidelines.ca/10.1155/2013/918216 PMC3673928 23748251

[17] Turk DC, Adams LM. Using a biopsychosocial perspective in the treatment of fibromyalgia patients. Pain Manag 2016;6(4):357. 10.2217/pmt-2016-0003 27301637

[18] Nielson WR, Merskey H. Psychosocial aspects of fibromyalgia. Curr Pain Headache Rep. 2001;5:330–37. 11403736 10.1007/s11916-001-0022-1

[19] Colmenares-Roa T, Huerta-Sil G, Infante-Casteñeda C, Lino-Pérez L, Alvarez- Hernández E, Peláez-Ballestas I. Doctor-patient relationship between individuals with fibromyalgia and rheumatologists in public and private health care in Mexico. Qual Health Res. 2016;26(12):1674–88. doi:10.1177/1049732315588742. 27578852

[20] Choy E, Perrot S, Leon T, Kaplan J, Petersel D, Ginovker A, Kramer E. A patient survey of the impact of fibromyalgia and the journey to diagnosis. BMC Health Serv Res. 2010;10:1–9. 20420681 10.1186/1472-6963-10-102 PMC2874550

[21] Egeli NA, Crooks VA, Matheson D, Ursa M, Marchant E. Patients’ views: improving care for people with fibromyalgia. J Clin Nurs 2008;17(11c):362–69. 26327419 10.1111/j.1365-2702.2008.02505.x

[22] Hasselroth R, Björling G, Faag C, Bose CN. “Can someone as young as you really feel that much pain? A survey on how people with fibromyalgia experience healthcare in Sweden. SAGE Open Nursing 2021;7:237796082110261–23779608211026145. doi:10.1177/23779608211026145. PMC8246575 34263029

[23] Skop MJ 2015. Maps of marginalization: exploring the healthcare experiences of men and women with fibromyalgia. [dissertation]. Ontario (Canada): Wilfred Laurier University. https://scholars.wlu.ca/etd/1708/

[24] McDaniel MM, Borgen WA, Buchanan MJ, Butterfield LD, Amundson NE. The philosophical underpinnings of the enhanced critical incident technique. Can J Couns Psycho. 2020;54(4):738–55. doi:10.47634/cjcp.v54i4.68139.

[25] Flanagan JC. The critical incident technique. Psychol Bull. 1954;51:327–58. 13177800 10.1037/h0061470

[26] Kemppainen JK. The critical incident technique and nursing care quality research. J Adv Nurs. 2000;32(5):1264–71. doi:10.1046/j.1365-2648.2000.01597.x. 11115012

[27] Woolsey LK. The critical incident technique: an innovative qualitative method of research. Can J Couns Psychol 1986;20(4):242–54. https://cjc-rcc.ucalgary.ca/article/view/59733

[28] Butterfield LD, Borgen WA, Maglio AT, Amundson NE. Using the enhanced critical incident technique in counselling psychology research. Can J Couns. 2009;43:265–82.

[29] Daraz L, MacDermid JC, Shaw L, Wilkins S, Gibson J. Experiences of women living with fibromyalgia: an exploratory story of their information needs and preferences. Rheumatol Rep. 2011;3:56–60.

[30] Sabik S. Fibromyalgia: stigmatization and its impact. J Appl Rehabil Couns. 2010;41(3):30–36. doi:10.1891/0047-2220.41.3.30.

[31] Waugh OC, Byrne DG, Nicholas MK. Internalized stigma in people living with chronic pain. J Pain. 2014;15:550–e1. 10.1016/j.jpain.2014.02.001 24548852

[32] Zotterman AN, Skär L, Olsson M, Söderberg S. Being in togetherness: meanings of encounters within primary healtcare [sic] setting [sic] for patients living with long-term illness. J Clin Nurs 2016;25(19-20):2854–62. 27383692 10.1111/jocn.13333

[33] Deslauriers S, Roy, J. S., Bernatsk S, Blanchard N, DE F, AM P, Fitzcharles M, Desmeules F, Perreault K. The burden of waiting to access pain clinic services: perceptions and experiences of patients with rheumatic conditions. BMC Health Serv Res 2021;21(160). doi:10.1186/s12913-021-06114-y. PMC7891805 33602224

[34] Michie S, Miles J, Weinman J. Patient-centredness in chronic illness: What is it and does it matter?. Patient Educ Couns 2003;51:197–206. 14630376 10.1016/s0738-3991(02)00194-5

[35] Chibnal JT, Tait RC, Gammack JK. Physician judgments and the burden of chronic pain. Pain Med 2018;19:1961–71. doi:10.1093/pm/pnx342. 29361153

[36] De Ruddere L, Goubert L, Vervoort T, Prkachin KM, Crombez G. We discount the pain of others when pain has no medical explanation. J Pain. 2012;13:1198–205. 23127294 10.1016/j.jpain.2012.09.002

[37] Tait RC, Chibnall JT, Kalauokalani D. Provider judgments of patients in pain: seeking symptom certainty. Pain Med. 2009;10:11–34. 18992039 10.1111/j.1526-4637.2008.00527.x

[38] Canadian Pain Task Force. 2020. Working together to better understand, prevent, and manage chronic pain: what we heard. A report by the Canadian Pain Task Force. [Internet]. October 2020. https://www.canada.ca/content/dam/hc-sc/documents/corporate/about-health-canada/public-engagement/external-advisory-bodies/canadian-pain-task-force/report-2020-rapport/report-2020.pdf

[39] Fitzcharles M, Cohen SP, Clauw DJ, Littlejohn G, Usui C, Häuser W. Nociplastic pain: towards an understanding of prevalent pain conditions. The Lancet (British Edution). 2021;397:209802110. 10.1016/S0140-6736(21)00392-5 34062144

[40] Sofaer-Bennett B. The role of counseling in chronic pain. In: Baranowski AP, Abrams P, Fall M, editors. Urogenital pain in clinical practice. Florida (USA): Taylor and Francis; 2008. p. 515–20.

